# Impact of a Structural Intervention to Address Alcohol Use Among Gay Bar Patrons in San Francisco: The PACE Study

**DOI:** 10.1007/s10461-017-1891-6

**Published:** 2017-08-24

**Authors:** Edwin D. Charlebois, Albert H. Plenty, Jessica Lin, Alicia Ayala, Jennifer Hecht

**Affiliations:** 1 Division of Prevention Science, Department of Medicine, Center for AIDS Prevention Studies, University of California, San Francisco, 550 15th St., 3rd Floor UCSF Mail Code 0886, San Francisco, CA 94158 USA; 2 STOP AIDS Project, San Francisco AIDS Foundation, 1035 Market Street, Suite 400, San Francisco, CA 94103 USA

**Keywords:** Structural intervention, Alcohol, HIV, MSM, Gay bars

## Abstract

We evaluated the impact on alcohol intake and blood alcohol concentration (BAC) of a multi-level structural intervention to increase the availability of free water, coupled with messaging on pacing alcohol intake and normative feedback of blood alcohol concentration in a convenience sample of gay bars in San Francisco. Participants (*n* = 1,293) were recruited among exiting patrons of four gay bars (two intervention bars and two control bars). Participants were surveyed on alcohol intake and BAC was measured by breathalyzer. Prior to the intervention there were no significant differences in baseline alcohol measures between intervention and control bars. Post-intervention there were significant differences on objective and subjective measures of alcohol consumption: 30% of intervention bar participants had BAC% levels over the legal driving limit (0.08%) compared to 43% of control bar participants, *p* < 0.0001 and 78% of intervention bar participants were above the AUDIT-C cut-off for hazardous drinking compared to 87% in control bars, *p* < 0.001.

## Introduction

An extensive body of research has linked alcohol consumption with unsafe sexual behavior, decreased safer sex negotiation, condom failure, and risks for HIV acquisition and transmission [1–13]. Multiple findings from the literature suggest that gay bar patrons are an important target group for alcohol and HIV risk interventions. Gay and bisexual men are more likely to frequent bars for socializing and the meeting of new sexual partners and more likely to continue heavy drinking later in life than the general population [14–16]. Green and Plant and others have observed that gay, bisexual and transgender people may have a greater reliance on using bars as social settings and places to meet partners than the general population, thus increasing their exposure to and risk of alcohol-related harms [16, 17]. In terms of risk behaviors, research has shown that Men-who-have-Sex-with-Men (MSM) whose social activity is centered around bars are more likely to engage in high-risk sexual behavior than those with non-bar centered socialization [18].

Structural interventions for HIV prevention that change the environments in which risk behavior occurs, without attempting to change knowledge, attitudes or social interaction patterns of the persons at risk have been suggested as potentially potent HIV prevention interventions [19–24]. Several structural interventions limiting alcohol availability have shown success at reducing either alcohol consumption or various problems associated with drinking in heterosexuals [25–28]. Likewise bar and alcohol server interventions have been suggested to reduce hazardous alcohol use among bar/restaurant patrons, but the data is conflicting [29–35]. However, few studies of structural interventions focusing on alcohol and MSM have been done to date.

We sought to develop and pilot test a multi-level structural intervention focused on gay bars to increase the availability of free water, coupled with in-bar messaging on using water to pace alcohol intake and individual normative feedback about blood alcohol concentration and evaluate the intervention’s impact on patron alcohol intake and blood alcohol concentration (BAC) among MSM in a convenience sample of gay bars in San Francisco, CA., USA.

## Methods

### Intervention Development and Description

This research took place in San Francisco, California, a city with a bustling bar scene with 357 bar liquor licenses in 2014, of which 57 were gay bars as identified by advertising or listing in gay travel guides [36]. The pacing alcohol consumption experiment (PACE) study was conducted in the heart of San Francisco’s gay neighborhood, the Castro, which has 13 gay bars located within a 2-block radius. Overall, an estimated 2.7 million alcoholic drinks are consumed per month by MSM in San Francisco with an average consumption of 40 alcoholic drinks per month per person [8].

We conducted a brief formative survey with 72 MSM community members to assess potential community acceptability and use of an intervention geared toward addressing alcohol use and about their experiences and opinions of San Francisco gay bars. Findings from the survey indicated; most men (62%) drink water as a tactic to decrease intoxication, 86% of men had asked for water at a bar, 69% had paid for water at some point, and 22% said water was between moderate and very hard to get at bars. From this preliminary investigation and from key informant interviews with bar owners we developed a multi-level structural intervention aimed at removing barriers to access and utilization of water in gay bars and promoting water use to pace alcohol intake.

The multi-level intervention was informed by the Social Ecological Model [37] which conceptualizes behaviors as influenced by concentric bands of influence representing the individual, interpersonal, organizational, community, and policy levels and by the empirically-validated PRECEDE model [38] that has shown that health promotion strategies are most effective when they combine: (1) “predisposing factors” comprised of knowledge, attitudes or beliefs that affect behavior; (2) “enabling factors” that facilitate change by making the behavior “easier”; and (3) “reinforcing factors” which include assessment and consequences of behaviors.

**Fig. 1:**
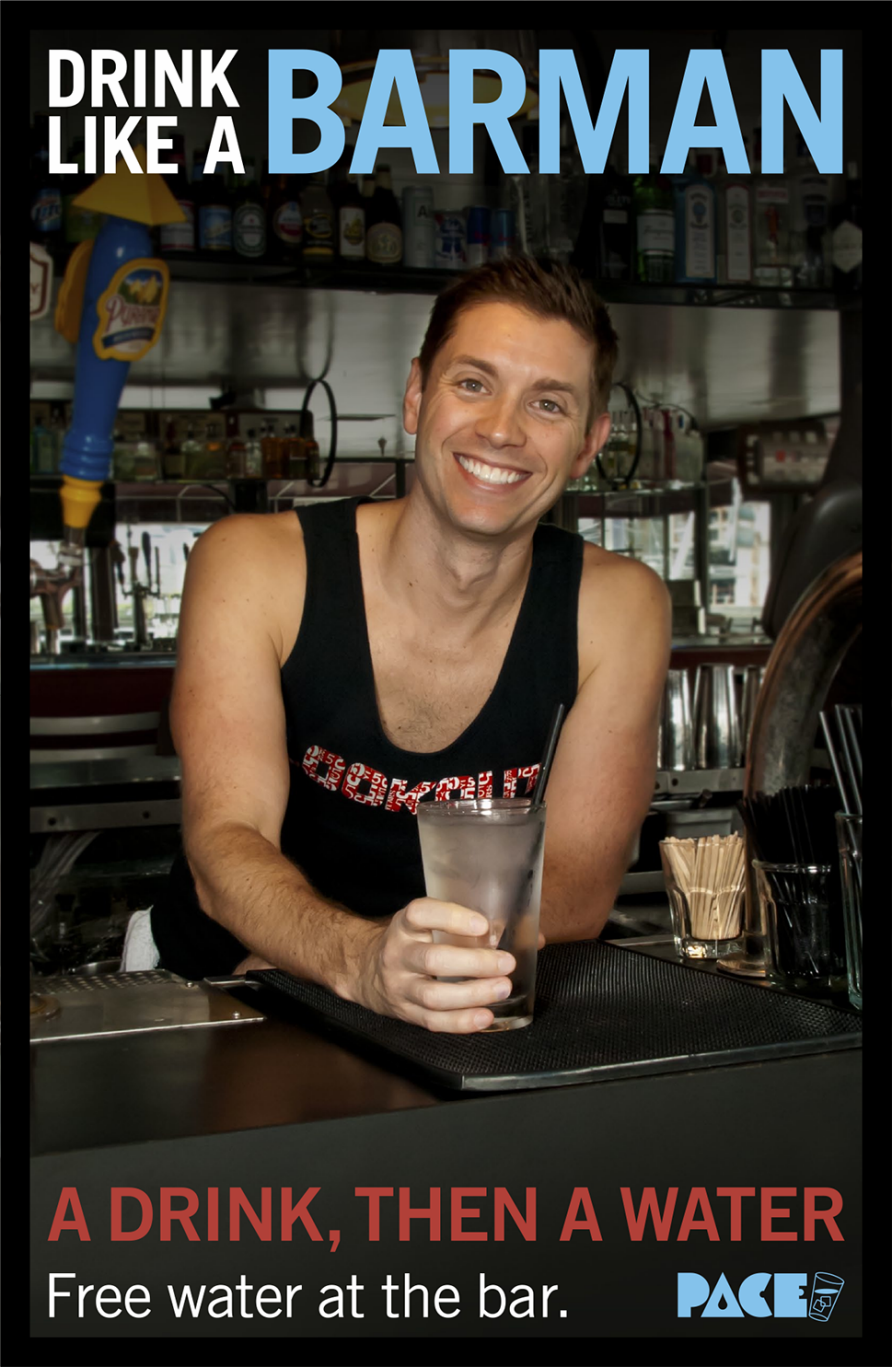
In bar media campaign poster featuring popular bartender

**Fig. 2:**
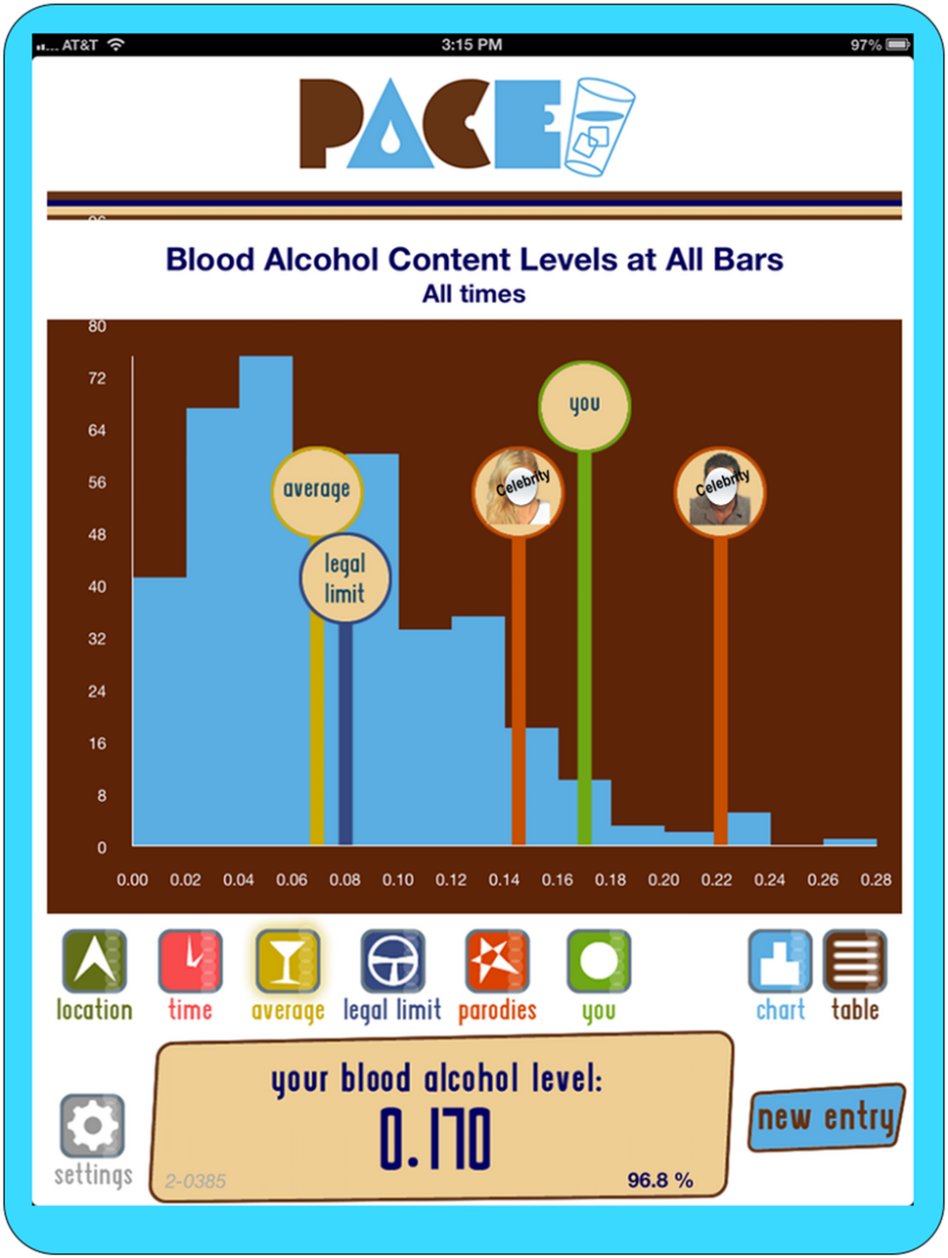
iPad normative feedback application screen

**Fig. 3:**
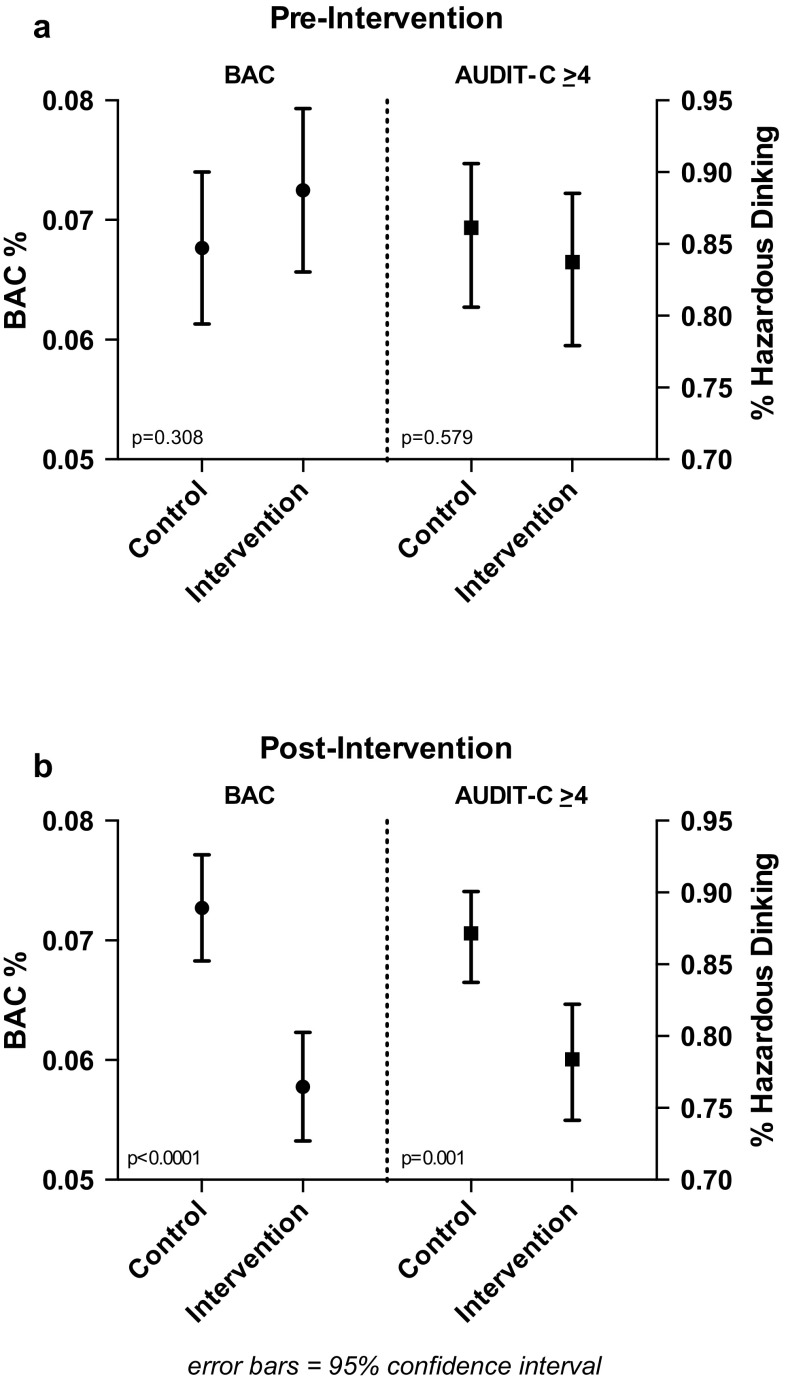
Pre-intervention (**a**) and post-intervention (**b**) comparison of mean blood alcohol concentration (BAC%) and percent hazardous drinking (AUDIT-C ≥ 4) by study arm

The intervention consisted of three main components (see Table 1); (1) a structural, physical component wherein freely accessible water was made available in the gay bar through installation of a centrally located filtered water tap or a free-standing, chilled bar-top water dispenser—*enabling*, (2) an environmental component of an in-bar media messaging campaign promoting the use of water for pacing alcohol intake using large posters of popular bartenders along with pacing and water attractiveness messaging—*predisposing* (see Fig. 1), and (3) an individual component of normative feedback of patron blood alcohol concentration (BAC) compared to the distribution of all other exiting gay bar patrons including the overall average of all bar patrons and indicators for legal driving limits displayed and individual’s BAC using an iPad app following administration of a hand-held breathalyzer test upon bar patron exit—*reinforcing* (see Fig. 2.).

**Table 1:** Multi-level structural alcohol intervention components

Level	Component	Mechanism
Structural	Freely available water	*Enabling*: removes physical and economic barriers to water access
Environmental	In-bar media campaign on using water to pace alcohol intake	*Predisposing*: promotion of water use for alcohol intake pacing using marketing techniques and popular opinion leaders
Individual	Normative feedback of blood alcohol concentration (BAC)	*Reinforcing*: comparison of self BAC level to that of peers serves as cue for reduction in drinking for those above the norm

Because installation of a free, self-service filtered water dispenser represents a significant alteration of the physical feature of bar establishments and the potential for bar patron flow disruption, we sought the cooperation and prior approval for study participation of four target bars from bar owners and managers. In our discussions with target bar owners and managers there was varied enthusiasm, but general acceptance of the idea of installing the free water dispenser, even among those bars that currently charge for water. Only one of eight approached bars declined the idea of participating. Following meetings and evaluation of bar clientele, we chose a convenience sample of four bars with approximately matched clientele based on age, location, and race/ethnicity of clients.

### Study Design

This developmental pilot was designed as a two-phase study with a 3-month (Sep 2012–Nov 2012) Pre-Intervention phase to confirm patron demographics matching and that there were no significant differences in the study primary outcome measures at baseline and a 18-month (Feb 2013–Aug 2014) Post-Intervention comparison phase where two gay bars received the intervention condition and the other two gay bars received the control condition. Of note both intervention bar and control bar participants received breathalyzer measurement coupled with normative feedback upon exiting the bars secondary to ethical and safety concerns related to BACs measured over the legal driving limit. The two bars selected to receive the full multi-level intervention were the first 2 bars to agree to the physical changes necessary to make water freely available.

### Subject Sampling and Eligibility

Subjects for evaluation in this study were sampled using a portal-exit sampling strategy [39, 40]. The study team recruited participants over the course of 2–3 h shifts conducted during days and times selected to have high clientele flow (between the hours of 5:00 pm–2:00 am on Thursday through Monday nights). Study staff members approached and attempted to recruit every patron exiting the bar’s main entrance (the portal) over the course of the 2–3 h shift. In order to maximize study participation, the field team included staff members who fit in with the gay bar atmosphere and would likely be perceived by bar attendees as approachable, nonjudgmental, and fun. Between 2–4 staff members were present during each recruitment shift to approach, screen, and survey potential participants.

Eligible participants were male-identified bar patrons over the age of 18 who had consumed at least one alcoholic beverage that night. Once eligibility was determined, study staff conducted verbal informed consent and offered a study information sheet to each participant. Participants received a small token of appreciation (e.g., candy, bottle-opener, toy of about $1–2 in value) and packet of safer sex supplies and referrals for their participation. Study subjects were deemed competent to engage in informed consent if they could repeat the study’s purpose and describe back to study staff the procedures that were to occur to them [41]. Individuals who were identified as too intoxicated to provide informed consent were offered assistance in finding safe transportation home and referral to other services. Institutional human subjects research approval was obtained for this study from the University of California at San Francisco, Committee on Human Research.

### Survey

Ten to twenty minute anonymous, interviewer-administered surveys were conducted with exiting patrons with handheld tablets (iPad, Apple, Inc., Cupertino, CA.) using the REDCap data collection system [42]. Survey items included subject demographics, motivations for choosing one bar over another (multiple choice + other, specify), prior bar attendance that evening, number and types of drinks consumed at current and prior bars, time spent in the bar that evening, the Alcohol Use Disorders Identification Test (AUDIT-C) [43] with hazardous drinking defined as an AUDIT-C score of ≥4, frequency of binge drinking (five or more drinks consumed at one setting), past sexual risk taking (defined as any condomless anal sex with a known or potentially HIV serodiscordant partner) and next intended destination (another bar, home, restaurant, other). In addition, participants sampled exiting intervention bars in the Post-intervention period were asked about exposure to the in-bar media campaign, their knowledge and use of installed water dispensers. All subjects in the Post-Intervention period (control and intervention bar patrons) were asked about how useful they found the normative feedback of their BAC compared to other bar patrons using a 5 point scale (‘Not at all’, ‘A little bit’, ‘Moderately’, ‘Quite a bit’, ‘Extremely’). Subjects were also asked about their intention to change drinking behaviors in response to the normative feedback (‘Yes, increase drinking’, ‘Yes, decrease drinking’, ‘No Intent to change’, and ‘I don't know’).

### Blood Alcohol Concentration (BAC) Measurement

Blood alcohol concentration (%) was measured using a handheld commercial breathalyzer testing unit (BACtrack S80 Pro; KHN Solutions, Inc., San Francisco, CA). These units are approved by the Department of Transportation for law-enforcement and were calibrated as recommended by the manufacturer. Participants were asked to swish and swallow water (provided by the study staff) at the start of survey participation in order to clear recent alcohol from their mouth and esophagus for breathalyzer testing at the end of the survey. Persons with measured BAC% above the legal California driving limit (0.08%) were offered assistance in finding safe transportation home or to their next destination.

### Statistical Analysis

The primary trial analysis was designed to be a comparison of the mean BAC% between individuals sampled from intervention bars and those sampled from control bars at the conclusion of the intervention period. Comparison testing during the Pre-Intervention period was carried out to confirm no significant differences were present in study outcome measures at baseline given the non-random assignment of intervention arm. Mean values, standard deviations, and 95% confidence intervals were calculated overall and by bar type (control/intervention) using standard equations [44]. To test for significant differences between the intervention and control bars for BAC% and other outcome measures we used Pearson *χ* ^2^ or Fisher’s Exact test, Student’s *t* test or non-parametric tests (where cell sizes went below accepted limits or distributions were non-normal). For comparisons of proportions of borderline statistical significance, single-sided tests were reflexively done with the assumed direction of the intervention resulting in the more favorable direction. In addition, we also performed multivariable linear modeling of measured BAC over the Pre- and Post-Intervention periods on the impact of intervention arm controlling for race and age using robust standard error calculation to account for clustering of measures within bars [45, 46]. Both a full model with all racial categories included and a best-fit model were calculated including indicators for time period (pre- and post-intervention) to account for temporal trend and interactions between time period and intervention bar status. All analyses were carried out using Stata version 14 (StataCorp LLC, College Station, TX).

## Results

A total of 1293 subjects participated in the bar exit survey and objective Blood Alcohol Concentration (BAC) measurement, 409 in the pre-intervention baseline period and 884 in the post-intervention evaluation period, with a total of 662 sampled from control bars and 631 sampled from intervention bars. Only 1% reported having been intercepted by the study team for interview a second time. Numbers of individuals in the study sample from the post-intervention period from each of the individual bars for the first matched control/intervention pair were 238 and 289, respectively and 171 and 186 for the second matched control/intervention bar pair. Overall participation rates for the portal exit-capture procedure varied nightly from 21 to 53% with a mean of 34% (95% CI 28.6–39.4%) and were in the usual range of published studies using bar exit-capture [47–50]. On average, patrons had spent 1.3 h in the exit bar (95% CI 1.21–1.34) with a range of <1–11 h and consumed a mean of 4.3 drinks (95% CI 4.0–4.7) with a range of 1–32 drinks. Forty percent of subjects reported that they had been at another bar previous to the sampled exit bar. Reasons for choice of one bar over another were: Where my friends go (32%), Pleasant crowd (21%), Events/Music (14%), Ambiance (13%), Location/convenience (10%), Drink specials (4%), Bartender (3%), Prices (2%), Strong drinks (1%), and Smoking area (<1%).

### Pre-Intervention Baseline Evaluation

Pre-Intervention baseline demographics, sexual risk taking, and alcohol consumption measures are presented in Table 2 for the control and intervention bars. Prior to the intervention there were no significant differences between our control and intervention bars on age, sexual risk taking, blood alcohol concentration, BAC ≥ 0.08, AUDIT-C score ≥ 4, or percent reporting binge drinking, though there were some minor differences by race between the intervention and control bars. The majority of our participants were white (57%) with 16% Latino, 10% Asian and less than 5% African American. The median age was below 35. Forty-three percent reported engaging in HIV transmission risk behavior (condomless anal sex with a potentially HIV serodiscordant partner at last sex). Of note, a very high percentage of participants (>80%) scored 4 or higher on the AUDIT-C, indicative of hazardous levels of drinking. On BAC testing, 42% were above the California legal limit for driving (0.08%).

**Table 2:** Pre-intervention subject characteristics and alcohol consumption

Pre-intervention	Control (*n* = 202)	Intervention (*n* = 207)	*p* value
Race			0.03 (overall)
American Indian/Alaska Native	1.0%	1.9%	0.69
Asian/P.I.	11.9%	8.7%	0.33
Black/African American	4.5%	4.8%	1.00
Latino	14.4%	17.9%	0.35
Multiracial	4.0%	12.1%	0.002
White	62.4%	52.2%	0.045
Not reported/not specified	2.0%	1.9%	1.00
Age			
Mean (IQR)	35.7 (28–43)	35.2 (28–42)	0.54
% reporting condomless, potentially serodiscordant anal sex with last partner	44.3%*	41.7%*	0.73
BAC%			
Mean (SD)	0.068% (0.046)	0.072% (0.049)	0.31
BAC% above CA. Driving limit			
0.0–0.07%	58.7%	57.2%	0.76
≥0.08% (above limit)	41.3%	42.8%	
AUDIT-C % hazardous drinking			
0–3	13.9%	16.3%	0.50
≥4 (hazardous drinking)	86.1%	83.7%	
Binge drinking at exit bar (5 or more)			
Yes	12.1%	10.9%	0.71
No	87.9%	89.1%	

* Sexual risk taking assessed in sub-sample of 88 control and 84 intervention subjects

**Table 3:** Post-intervention subject characteristics and alcohol consumption

Post-intervention	Control (*n* = 460)	Intervention (*n* = 424)	*p* value
Race			0.10 (overall)
American Indian/Alaska Native	2.6%	1.9%	0.51
Asian/P.I.	6.1%	8.0%	0.30
Black/African American	3.7%	6.1%	0.12
Latino	9.6%	12.0%	0.28
Multiracial	4.8%	6.4%	0.31
White	72.6%	63.7%	0.005
Not reported/not specified	0.6%	1.9%	0.13
Age			
Mean (IQR)	38.7 (29–46)	35.8 (28–42)	<0.0001
% reporting condomless, potentially serodiscordant anal sex with last partner	41.2%*	43.0%*	0.64
BAC%			
Mean (SD)	0.073% (.048)	0.058% (.047)	<0.0001
BAC % above CA. Driving limit			
0.0–0.07%	57.4%	70.7%	<0.0001
≥0.08% (above limit)	42.6%	30.0%	
AUDIT-C % hazardous drinking			
0–3	12.9%	21.6%	0.001
≥4 (hazardous drinking)	87.1%	78.4%	
Binge drinking at exit bar (5 or more)			
Yes	8.1%	4.8%	0.055
No	91.9%	95.2%	(0.033 1-sided)

* Sexual risk taking assessed in sub-sample of 354 control and 330 intervention subjects

### Post-Intervention Comparison

Post-Intervention subject demographics, sexual risk taking, and alcohol consumption measures are presented in Table 3 by control and intervention arm. A minor, but statistically significant difference in the mean age of the subjects between the intervention and control bars (less than 3 years) was seen. After the intervention, we found significantly lower levels of BAC%, and hazardous levels of drinking by AUDIT-C in intervention bar patrons compared to control bar patrons (see Fig. 3). For measured BAC, we found that 43% of control bar participants were over the 0.08% California legal driving limit compared to 30% of patrons exiting intervention bars (*p* = 0.001). Post-intervention mean AUDIT-C scores also differed significantly between the intervention and control bars with a mean score of 5.6 for intervention subjects versus 6.3 for control subjects (*p* < 0.0001) in contrast to finding no significant differences pre-intervention. In addition, we found an almost 50% lower percentage of participants reporting binge drinking that evening at the intervention bars compared to the control bars (4.8% intervention vs. 8.1% control, *p* = 0.055, = 0.033 1-sided) post intervention. No difference was seen in the percent of subjects reporting HIV transmission risk behavior with their last sex partner.

In multivariate modeling of measured BAC controlling for age, race, clustering and calendar time period (pre- vs. post-intervention), the intervention arm was significantly associated with lower BACs with a linear component effect of −0.0156 (95% CI −0.009 to −0.022, *p* < 0.001) and significant effects for African American race of −0.014 (95% CI −0.0005 to −0.023, *p* = 0.002) and age of −0.0003 per year older (95% CI−0.00007 to −0.0006, *p* = 0.013), with a constant of 0.080 (95% CI 0.069–0.090, *p* < 0.001). Due to collinearity in the data, only an interaction effect for intervention bar status in the pre-intervention could be estimated (linear effect of 0.02, 95% CI 0.009 to 0.031, *p* < 0.001). No significant calendar time effect indicative of a secular trend (pre- vs post-intervention) was seen in the adjusted model (linear effect of 0.02, 95% CI −0.002 to 0.014, *p* = 0.143).

### Exposure to and Uptake of Intervention Components

Among subjects exiting intervention bars, 21% (95% CI 17.5–25.6%) reported seeing an in-bar media campaign poster that evening. Twenty-eight percent (95% CI 24.5–33.5%) reported seeing the free water dispenser during their time in the bar and among those, 25% (95% CI 18.2–34.8%) reported using the water dispenser at that bar visit. When asked about the usefulness of the BAC normative feedback, 48.5% (95% CI 43.8–53.2%) of control bar subjects thought the normative feedback was “quite a bit’ or “extremely’ useful compared to a higher proportion in intervention bar subjects (55.2%, 95% CI 50.3–60.0%, p = 0.046). Overall, 10.0% of control and intervention subjects reported that they intended to decrease their drinking in response to seeing their BAC and the normative feedback and this differed significantly by intervention condition with a higher proportion of intervention bar subjects reporting that they intended to decrease their drinking 12.8% (95% CI 9.8–16.4%) compared to control bar subjects 6.8%, 95% CI 4.7–9.6%, *p* = 0.005). Among those with BACs greater than the overall bar mean (>0.065%), intentions to decrease drinking were 16.5% (95% CI 11.0–23.2%) for intervention bar subjects and only 7.3% (95% CI 4.3–11.5%) for control bar subjects (*p* = 0.005).

## Discussion

In a developmental pilot test of a multi-level structural alcohol intervention in gay bars consisting of the provision of free water, coupled with a bar media campaign on the use of water to pace alcohol intake and normative feedback of patron blood alcohol concentration compared to other bar patrons, we found significant significantly lower BAC% and AUDIT-C scores and lower levels of binge drinking at intervention bars compared to control bars. Both objective measures of alcohol consumption (measured BAC% by breathalyzer) and subjective measures of alcohol intake and misuse (self-report AUDIT-C and binge drinking) showed significantly lower levels of alcohol and hazardous drinking among intervention bar subjects compared to control bars subjects post-intervention whereas no differences were observed between the bars pre-intervention. The post-intervention effect on BAC was present in both of the intervention bars equally, with reductions in the mean BAC of 0.013 and 0.017 relative to each bar’s pre-intervention mean (*p* < 0.01 for both). The strength and consistency of the results are highly supportive of a distinct beneficial intervention effect among gay bar patrons exposed to the multi-level intervention.

As a proof of concept and feasibility trial this study has demonstrated that it is possible to engage with gay bar venues as sites for alcohol as well as HIV risk reduction interventions. These bars already play a central role in HIV prevention in the gay community as locations for distribution of condoms and safer sex messages and as social spaces for fundraisers for HIV care and prevention agencies. Given the growing community and public health system awareness of alcohol misuse as a contributing factor in new HIV infections and the significant potential of structural interventions to produce lasting, efficient and cost-effective benefits, this research strongly points towards the feasibility and potential impact of the tested multi-level intervention to address alcohol use among MSM in gay bar settings.

Indeed, structural alcohol interventions of this type are likely to be effective in responding to two identified priority areas for HIV prevention in San Francisco and elsewhere; (1) alcohol as a driver of the HIV epidemic and (2) the high risk of HIV infection among young MSM. The San Francisco Department of Public Health and the HIV Prevention Planning Council have officially identified alcohol as “driver” of the local HIV epidemic by virtue of alcohol’s prevalent heavy use (10% or higher in the population) combined with evidence showing an independent association of alcohol doubling the risk of HIV acquisition [51]. Gay bar venues may be an excellent intervention point for modifying alcohol associated HIV risk among MSM. Evidence supporting this supposition is that among young MSM 18–29 years old in San Francisco, frequent bar attendance has been shown to be significantly associated with heavy alcohol use and binge drinking (>5 drinks per episode, OR 2.17, *p* < 0.001) [52] compared to those with less frequent bar attendance. This finding is echoed among MSM of all ages in San Francisco in the 2011 NHBS survey where 61% of MSM reported binge drinking in the past month among those sampled from venues serving alcohol compared to 31% reporting binge drinking among MSM sampled from venues not serving alcohol (*p* < 0.0001) [8]. The finding from this current study of a significant age association with BAC with younger MSM having higher levels of blood alcohol particularly points to the potential utility of this intervention in addressing alcohol misuse and associated HIV risks among young MSM.

Overall, the study found high levels of alcohol use and misuse in a context of significant sexual risk among gay bar patrons, echoing concerns and opportunities for intervention with this venue-associated population of MSM. Although the study was able to observe significant reductions in alcohol intake and misuse in response to the intervention, observation of reductions in subsequent HIV transmission risks associated with the alcohol impact were more challenging to see. We did not see any reductions in reported condomless, potentially serodiscordant anal sex with last partner at the time of exit from the bar in the post-intervention period. It is likely that retrospective recall of transmission risk at last sex may not be the correct temporal link to behavior change in response to an intervention effect that has just occurred (currently less intoxicated). Future evaluations of the intervention effect on subsequent sexual risk should attempt to measure sexual risk behavior on the evening following bar exit in a follow-up cohort design, something that was not possible with the current study’s anonymous design.

The study has a number of limitations. First, this developmental study was designed as a non-randomized, contemporaneously controlled trial of a small number of gay bar venues. The non-random choice of intervention bar status, although motivated by practical concerns, could have introduced bias accounting for some of the observed differences between the control and intervention bars. Arguing against this possibility is the similarity in the study’s primary outcome measures pre-intervention and the degree to which the bars were well-matched on clientele and alcohol intake patterns. Second, use of the portal exit-capture strategy for subject selection was associated with a participation rate less than 50%, calling into question the representativeness of the study sample. Community level effectiveness trials, particularly venue-based studies, are often challenged by recruitment strategies and the response rates seen in this study are in line with other studies of heterosexual bars in the alcohol research field. Although the lower participation rate may make the study estimates biased in the absolute, the relative comparisons between the control bar and intervention bar subjects remain valid given that we saw no evidence of differential participation rates or characteristics by intervention arm status. Likewise, because of the exclusion of potential study subjects who were too intoxicated to participate in informed consent, the estimates of BAC and binge drinking prevalence are likely to be conservative estimates and would have been higher if those intoxicated persons had been included in the study sample.

In conclusion, we observed a significant beneficial intervention effect of lower levels of blood alcohol concentration (BAC) and lower levels of reported hazardous and binge drinking among MSM in San Francisco gay bars in response to a multi-level, structural alcohol intervention consisting of freely accessible water, in-bar media messaging campaign promoting the use of water for pacing alcohol intake and normative feedback of patron BAC compared other gay bar patrons. Further research expanding and adapting this intervention to other cities outside of San Francisco is warranted.
